# The effect of divalent ions on L-α-phosphatidylcholine from egg yolk monolayers at the air/water interface

**DOI:** 10.1007/s00775-017-1495-7

**Published:** 2017-10-19

**Authors:** Aneta D. Petelska, Monika Naumowicz

**Affiliations:** Institute of Chemistry, University of Bialystok, 15-443 Bialystok, Ciolkowskiego 1K, 15-245 Białystok, Poland

**Keywords:** L-α-Phosphatidylcholine, Divalent ions, Complexes formation equilibria, Monolayer, Langmuir trough

## Abstract

The Langmuir monolayers of L-α-phosphatidylcholine from egg yolk were studied by Wilhelmy method. The surface pressure versus molecular area isotherm of lipid on pure water and different subphase (with a presence of divalent ions: Sr^2+^, Cd^2+^, Ba^2+^, Pb^2+^) was obtained. The limiting area of the isotherms depends on the presence of subphase ions. The addition of divalent ions to the subphase stabilized the monolayers and increased the limiting areas of the monolayer. During the compression in monolayer complexes of 1:1 and 2:1 stoichiometry between L-α-phosphatidylcholine from egg yolk and divalent ions are formed. We used the equilibrium theory to describe the behavior of monolayer components at the air/water interface. An equilibrium theory to describe the behavior of monolayer components at the air/water interface was developed in order to obtain the stability constants and area occupied by one molecule of LMe^2+^ or L_2_Me^2+^ complexes, and complex formation energy (Gibbs free energy) values. These mathematically derived and experimentally confirmed values are of great importance for the interpretation of phenomena occurring in lipid monolayers and bilayers.

## Introduction

For many years, researchers have used Langmuir films as model systems of biological membranes [1]. Amphiphilic monolayers (surface films formed at the air/water interface) are useful for studying mechanisms of biophysical and biochemical phenomena in living cells. These monolayers can provide important knowledge regarding the properties of thin amphiphilic arrangements (e.g., fatty acids, lipids, proteins, and mixed films) in agricultural, pharmaceutical, and food-science applications [2, 3]. Characteristic properties of molecules at the air/water interface are generally characterized by *π*–*A* isotherms, where the surface pressure of the monolayer (*π*) is a function of surface area per molecule (*A*). The molecular limiting areas are obtained by extrapolation of the steep linear portion(s) of the *π* vs. *A* curves to *π* = 0 [4].

**Fig. 1:**
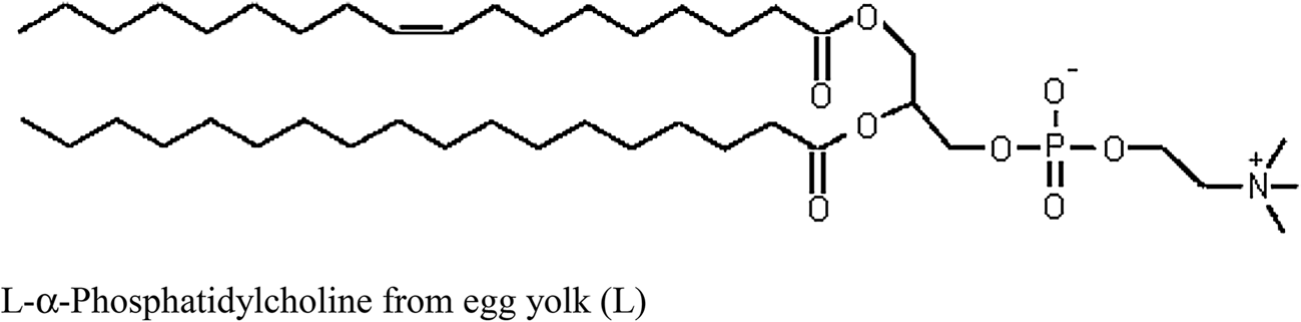
The chemical structure of L-α-phosphatidylcholine from egg yolk molecule

L-α-Phosphatidylcholine from egg yolk (Fig. 1) monolayer models have been used to reconstruct various biophysical processes in biological membranes [2, 3, 5–8]. Physicochemical properties of these monolayers depend on the three-dimensional structures of lipid molecules, their packing density at the interface, the pH of the subphase, and its ionic composition and concentration [3, 5]. Monolayer–subphase interactions can be widely varied by changing the head and tail parts of the molecule (e.g., by precisely varying the length of a hydrocarbon chain), or by changing the pH [9, 10] or ion content of the subphase [11–22]. Many interesting information on monolayers is also provided by Chifu’s group studies about thermodynamics of equilibria in monolayers at various surface pressures, including the collapse pressure [23, 24].

Previous investigations of interactions of divalent cations [18, 21, 25–28] (e.g., Ca^2+^, Mg^2+^, and Zn^2+^) with phospholipid membranes indicated the formation of well-defined chemical complexes whose stoichiometries depended on the type of metal cation, type of lipid, phase state, and water activity. For example, one Ca^2+^ ion coordinated with two 1-palmitoyl-2-oleoylphosphatidylcholine molecules in the fluid phase under the excess water condition [25]. Effects of highly concentrated salt solutions of marine-relevant cations (Na^+^, K^+^, Ca^2+^, Mg^2+^) on Langmuir monolayers of dipalmitoylphosphatidylcholine were investigated by surface pressure–area isotherms, Brewster angle microscopy (BAM), and infrared reflection–absorption spectroscopy (IRRAS) [29]. Using surface potentiometry, Allen and coworkers [30] investigated the surface potential of model zwitterionic dipalmitoylphosphatidylcholine monolayers on concentrated aqueous chloride solutions of alkali (Na^+^, K^+^) and alkaline (Ca^2+^, Mg^2+^) earth cations prevalent in the marine environment. Other researchers studied effects of divalent cations (Ca^2+^, Mg^2+^, Ni^2+^, and Zn^2+^) on zwitterionic phospholipid monolayers at the air/water interface by surface pressure–area isotherms and in situ X-ray scattering [31].

Metal ions have unique chemical properties that allow these ions to play diverse roles in cellular biochemistry [32]. There are many reasons for studying effects of divalent ions on the interactions between charged surfaces and for characterizing ionizable model surfaces [33]. Langmuir monolayers at the air/water interface are a suitable system for studying binding between a biological component (e.g., DNA) and zwitterionic lipids [34]. Such experiments could include investigations of the effects of divalent ions, which are needed for favorable adsorption of DNA to the monolayer surface. Apart from fundamental interest, practical applications of cation binding to L-α-phosphatidylcholine membranes have received special attention [11, 12, 27, 34]. Recently, researchers showed that interactions between DNA and zwitterionic lipids are strongly enhanced by electrostatic interactions in the presence of Ca^2+^. Therefore, they might serve as suitable vectors for DNA transfection, as zwitterionic lipids are nontoxic and biocompatible, in contrast to most cationic lipids [34]. Interactions between phospholipid membranes and ions also play key roles in many biological processes, such as neural signal transduction [21] and membrane fusion [25].

The interactions studied may have implications related to the toxic and physiologic effects of heavy metals on living tissues. The interfacial interactions between stearic acid monolayers and the heavy metal ions, zinc, cadmium, and mercury have been investigated as part of a broader study investigating the effects of heavy metals on model monolayers simulating the human alveolus [35]. Authors are postulated that the interaction occurs in a 2:1 ratio of fatty acid to metal ion. The activity of the Hg^2+^ ion for causing changes in the surface properties of the films was much less than that observed for Zn and Cd [35].

The purpose of this work was to continue the systematic study by Petelska and coworkers [17, 19–21] of the effects of monovalent and divalent ions on L-α-phosphatidylcholine from egg yolk monolayers. Specifically, we sought to examine the possible effects of divalent ions on L-α-phosphatidylcholine from egg yolk monolayer properties and the molecular interactions between L-α-phosphatidylcholine from egg yolk (denoted by L) and divalent ions (denoted by Me^2+^: Sr^2+^, Ba^2+^, Cd^2+^, Pb^2+^). Despite this low concentration, heavy metals still had an observable effect on the L-α-phosphatidylcholine from egg yolk compression isotherm. The interactions studied may have implications related to the toxic and physiologic effects of heavy metals on living tissues.

In this paper, we present evidence for the formation of LMe^2+^ and L_2_Me^2+^ complexes at the air/water interface and report their stability constants, areas occupied by one complexes molecule and complex formation energies. A new, simple and very interesting method proposed by us can be used with success for the determination of the parameters used to describe any 1:1 and 2:1 lipid–divalent ion complexes. In our opinion, this information will be very helpful in understanding the transmembrane transport mechanisms for ions, toxins, and drugs.

## Theory

The individual components L-α-phosphatidylcholine from egg yolk (denoted by L) and divalent ions (denoted by Me^2+^: Sr^2+^, Cd^2+^, Ba^2+^, Pb^2+^) can form complexes in a mixed two-component monolayer on a free electrolyte surface. In a mixed monolayer at the air/water interface 1:1 (LMe^2+^) and 2:1 (L_2_Me^2+^) complexes are formed. The equilibria of such a system are described by the complexation reaction presented below [17, 20].

1 $$ {\text{L}} + {\text{Me}}^{2 + } \Leftrightarrow {\text{LMe}}^{2 + } $$

2 $$ {\text{LMe}}^{2 + } + {\text{L}} \Leftrightarrow {\text{L}}_{2} {\text{Me}}^{2 + } $$

and the equilibrium state of the discussed system might be described by the system of equation:

3 $$ K_{1} = \frac{{c_{{{\text{LMe}}^{2 + } }} }}{{c_{\text{L}} \times c_{{{\text{Me}}^{2 + } }} }}, $$

4 $$ K_{2} = \frac{{c_{{{\text{L}}_{ 2} {\text{Me}}^{2 + } }} }}{{c_{{{\text{LMe}}^{2 + } }} \times c_{\text{L}} }}, $$

5 $$ c_{\text{L}} A_{\text{L}} + c_{{{\text{LMe}}^{2 + } }} A_{{{\text{LMe}}^{2 + } }} + c_{{{\text{L}}_{2} {\text{Me}}^{2 + } }} A_{{{\text{L}}_{2} {\text{Me}}^{2 + } }} = 1, $$

6 $$ c_{\text{L}} + c_{{{\text{LMe}}^{2 + } }} + 2c_{{{\text{L}}_{2} {\text{Me}}^{2 + } }} = C, $$

where $c_{\text{L}} ,c_{{{\text{LMe}}^{2 + } }} ,c_{{{\text{L}}_{2} {\text{Me}}^{2 + } }}$ (mol m^−2^) are the surface concentrations of components L, LMe^2+^, L_2_Me^2+^; $c_{{{\text{Me}}^{2 + } }}$ (mol m^−3^) is the concentrations of Me^2+^ ions; $A_{\text{L}} ,A_{{{\text{LMe}}^{2 + } }} ,A_{{{\text{L}}_{2} {\text{Me}}^{2 + } }}$ (m^2^ mol^−1^) are the surface areas occupied by 1 mol of components L, LMe^2+^, L_2_Me^2+^; $K_{1}$ (m^3^ mol^−1^) $K_{2}$ (m^2^ mol^−1^) are stability constant of LMe^2+^ and L_2_Me^2+^ complexes; *C* (mol m^−2^) is the total surface concentration.

Elimination $c_{\text{L}} ,c_{{{\text{LMe}}^{2 + } }} ,c_{{{\text{L}}_{2} {\text{Me}}^{2 + } }}$ parameter from the set of Eqs. (3)–(6) yields the basic equation presented below [17, 20]:

7 $$ y = m_{1} x_{1} + m_{2} x_{2} + m_{3} x_{3} + m_{4} x_{4} + m_{5} x_{5} + b, $$

where

$$ y = C^{2} c_{{{\text{Me}}^{2 + } }} $$

$$ m_{1} = K_{1} K_{2}^{ - 1} A_{{{\text{L}}_{2} {\text{Me}}^{2 + } }}^{ - 2} A_{{{\text{LMe}}^{2 + } }} \left( {2A_{{{\text{LMe}}^{2 + } }} - A_{{{\text{L}}_{2} {\text{Me}}^{2 + } }} } \right) $$

$$ x_{1} = Cc_{{{\text{Me}}^{2 + } }}^{2} $$

$$ m_{2} = K_{2}^{ - 1} A_{{{\text{L}}_{2} {\text{Me}}^{2 + } }}^{ - 2} \left[ {4K_{2} A_{{{\text{L}}_{2} {\text{Me}}^{2 + } }} + A_{\text{L}} \left( {2A_{{{\text{LMe}}^{2 + } }} - A_{{{\text{L}}_{2} {\text{Me}}^{2 + } }} } \right) + A_{{{\text{LMe}}^{2 + } }} \left( {2A_{\text{L}} - A_{{{\text{L}}_{2} {\text{Me}}^{2 + } }} } \right)} \right] $$

$$ x_{2} = Cc_{{{\text{Me}}^{2 + } }} $$

$$ m_{3} = K_{1}^{ - 1} K_{2}^{ - 1} A_{{{\text{L}}_{2} {\text{Me}}^{2 + } }}^{ - 2} A_{{{\text{L}}^{{ ^{ - } }} }} \left( {2A_{\text{L}} - A_{{{\text{L}}_{2} {\text{Me}}^{2 + } }} } \right) $$

$$ x_{3} = C $$

$$ m_{4} = - K_{1} K_{2}^{ - 1} A_{{{\text{L}}_{2} {\text{Me}}^{ 2+ } }}^{ - 2} \left( {2A_{{{\text{LMe}}^{2 + } }} - A_{{{\text{L}}_{2} {\text{Me}}^{2 + } }} } \right) $$

$$ x_{4} = c_{{{\text{Me}}^{2 + } }}^{2} $$

$$ m_{5} = - K_{2}^{ - 1} A_{{{\text{L}}_{2} {\text{Me}}^{2 + } }}^{ - 2} \left[ {4K_{2} + \left( {2A_{\text{L}} - A_{{{\text{L}}_{2} {\text{Me}}^{2 + } }} } \right) + \left( {2A_{{{\text{LMe}}^{2 + } }} - A_{{{\text{L}}_{2} {\text{Me}}^{2 + } }} } \right)} \right] $$

$$ x_{5} = c_{{{\text{Me}}^{2 + } }} $$

$$ b = - K_{1}^{ - 1} K_{2}^{ - 1} A_{{{\text{L}}_{2} {\text{Me}}^{2 + } }}^{ - 2} \left( {2A_{\text{L}} - A_{{{\text{L}}_{2} {\text{Me}}^{2 + } }} } \right). $$

The $K_{1} ,K_{2} ,A_{\text{L}} ,A_{{{\text{LMe}}^{2 + } }} ,A_{{{\text{L}}_{ 2} {\text{Me}}^{2 + } }}$ parameters were calculated from equation presented below:

8 $$ A_{\text{L}} = \frac{{ - m_{3} }}{b} $$

9 $$ A_{{{\text{LMe}}^{2 + } }} = \frac{{ - m_{1} }}{{m_{4} }} $$

10 $$ A_{{{\text{L}}_{2} {\text{Me}}^{2 + } }} = 2\frac{{A_{\text{L}} m_{4} - A_{{{\text{LMe}}^{2 + } }} b}}{{m_{4} - b}} $$

11 $$ K_{1} = - \frac{{2A_{{{\text{LMe}}^{2 + } }} - A_{{{\text{L}}_{2} {\text{Me}}^{ 2+ } }} }}{{m_{4} K_{2} A_{{{\text{L}}_{2} {\text{Me}}^{2 + } }}^{2} }} $$

12 $$ K_{2} = - \frac{{\left( {2A_{\text{L}} - A_{{{\text{L}}_{2} {\text{Me}}^{2 + } }} } \right) + \left( {2A_{{{\text{LMe}}^{2 + } }} - A_{{{\text{L}}_{2} {\text{Me}}^{2 + } }} } \right)}}{{4 + m_{5} A_{{{\text{L}}_{2} {\text{Me}}^{2 + } }}^{2} }}. $$

The $K_{1} ,K_{2} ,A_{\text{L}} ,A_{{{\text{LMe}}^{2 + } }} ,A_{{{\text{L}}_{2} {\text{Me}}^{2 + } }}$ parameters were calculated from Eqs. (8)–(12) and described the complexes formed at the air/water interface in mixed monolayers (presented in Table 1).

The obtained parameters describing the complexes may be used to calculate theoretical points (presented on Fig. 3) using the equation presented below (agreement between the theoretical and experimental values implies that the system is well described by the above equations) [17, 20]:

13 $$ K_{1} K_{2} A_{{{\text{L}}_{2} {\text{Me}}^{2 + } }} c_{{{\text{Me}}^{2 + } }} c_{\text{L}}^{2} + \left( {K_{1} A_{{{\text{LMe}}^{2 + } }} c_{{{\text{Me}}^{2 + } }} + A_{\text{L}} } \right)c_{\text{L}} - 1 = 0, $$

where the surface concentration of L form was calculated from Eq. (13)

14 $$ c_{\text{L}} = \frac{{ - K_{1} A_{{{\text{LMe}}^{2 + } }} c_{{{\text{Me}}^{2 + } }} - A_{\text{L}} + \sqrt \Delta }}{{2K_{1} K_{2} A_{{{\text{L}}_{2} {\text{Me}}^{2 + } }} c_{{{\text{Me}}^{2 + } }} }} $$

and surface concentration of other forms LMe^2+^ and L_2_Me^2+^ were calculated according to Eqs. (15) and (16):

15 $$ c_{{{\text{LMe}}^{2 + } }} = K_{1} c_{\text{L}} c_{{{\text{Me}}^{2 + } }} $$

16 $$ c_{{{\text{L}}_{2} {\text{Me}}^{2 + } }} = K_{2} c_{{{\text{LMe}}^{2 + } }} c_{\text{L}} . $$

The total surface concentration of L-α-phosphatidylcholine from egg yolk membrane and divalent Me^2+^ ions was calculated from the sum of surface concentration of all forms presented in air/water interface (L, LMe^2+^ and L_2_Me^2+^; presented in Fig. 3) according to Eq. (6) [17, 20].

The L-α-phosphatidylcholine from egg yolk–divalent ions complex formation energies were calculated from Eq. (17):

17 $$ - \log K = \frac{{\Delta G^{ 0} }}{2.3RT} $$

where $K$ (m^2^ mol^−1^) is the stability constant of L-α-phosphatidylcholine from egg yolk–divalent ions complex; $\Delta G^{ 0}$ (J mol^−1^) is the L-α-phosphatidylcholine from egg yolk–divalent ions complex formation energy; $R$(J mol^−1^ K^−1^) is gas constant; $T$ (K) is the temperature.

## Materials and methods

### Film-forming materials

The L-α-phosphatidylcholine from egg yolk was purchased from Sigma and used in the experiment as received; it had the following fatty acid composition 6:0∼33%; 18:0∼4%; 18:1∼30%; 18:2∼14%; 20:4∼4%. The declared purity of the lipid was 99%.

### Spreading solvent

1-Chloropropane (Aldrich) was employed as a spreading solvent for L-α-phosphatidylcholine from egg yolk. The solvent was of > 98% purity, used without further purification. The spreading of the solvent did not alter the surface tension of the subphase, indicating the absence of impurities with surface activity. Solutions were prepared by dissolving of L-α-phosphatidylcholine from egg yolk in 1-chloropropane at a concentration of 1 mg cm^−3^. Solutions were stored at 4 °C until use.

### Subphases

Triple-distilled water (pH 7; second distillation performed over KMnO_4_ and KOH, both from POCh (Polish Chemical Reagents) to remove organic impurities), containing various electrolyte, was used as a subphase for the L-α-phosphatidylcholine from egg yolk monolayer. Electrolyte solutions (concentration range 5.0 × 10^−5^–5.0 × 10^−3^ mol dm^−3^) were prepared from triple-distilled water and strontium chloride (SrCl_2_, 99%), barium chloride (BaCl_2_, 99%), cadmium chloride (CdCl_2_, 99.9%), and lead nitrate (Pb(NO_3_)_2_, 99%), purchased from Sigma-Aldrich (St. Louis, MO, USA). The electrolyte was of p.a. purity and used without further purification. They contained no impurities with surface activity, since the subphases showed zero surface pressure before the spreading of the L-α-phosphatidylcholine from egg yolk.

### Work conditions and experimental procedure

The desired amount of surfactant solution was placed on the subphase by means of a Hamilton micro-syringe. An overall waiting time of 10–15 min was allowed for evaporation of the spreading solvent and the start of the experiment. The monolayer was continuously compressed to obtain the *π*–*A* isotherms using the glass barrier. The glass material allowed lipid molecules to pass under the barrier. This innovation considerably improved the reproducibility of the results [36]. The equilibrium between monolayer and the subphase was established rather rapidly, allowing us to record an isotherm in several minutes (between 10 and 30 min).

No chemical degradation of the investigated L-α-phosphatidylcholine from egg yolk was observed, neither in the spreading solutions nor in the monolayer, in time intervals equal to the duration of the experiments.

The compression isotherms were recorded by the Wilhelmy methods. Surface tension was measured at the water/air interface at 22 °C by using a homemade computer-controlled apparatus, as described previously [36]. It consists of the a 9000 Nima tensiometer, a Teflon trough of 648 cm^2^ surface area, a thin glass plate, a glass barrier, a moving glass barrier system and a control unit of tensiometer. The dependence of surface tension on monolayer surface area was recorded by the ST9002 Nima computer program. The glass barrier was moving at 0.03 cm s^−1^ velocity [36]. Surface tension results were expressed as surface pressure area per molecule (π–A) isotherms. The Nima ST9002 computer program was used to calculate the surface pressure of the monolayer (*π*) as a function of surface area per molecule (*A*): *π* = *γ*_0_ − *γ* = *f*(*A*), where *γ*_0_ is the surface tension of the bare air/water interface, and *γ* is the surface tension of the lipid-covered surface.

Before each run, Teflon trough (trough size 648 cm^2^) was washed and rinsed with purified water. The subphase surface was cleaned each time just before the measurement by suction with a vacuum pump until the results of surface tension measurements with the ST9000 Nima tensiometer were constant and equal to the surface tension value of pure water at 22 °C (about 72 mN m^−1^). Before use, all glass in contact with the samples was cleaned with chromic acid and was exhaustively rinsed with highly purified water [36].

The system was enclosed in an acrylic box to minimize water evaporation, ensure high humidity, and avoid contamination. Reported values are highly reproducible and represent the average of at least five experiments. Standard deviations for surface area measurements were less than 1%.

## Results and discussion

In this paper, we present surface tension measurements of L-α-phosphatidylcholine from egg yolk monolayers obtained using a Langmuir method as a function of divalent Me^2+^ ion concentration. We obtained evidence for the formation of L-α-phosphatidylcholine from egg yolk–divalent ion complexes at the air/water interface and developed a system of equations to describe formation of these complexes, which we used to calculate stability constants.

**Fig. 2:**
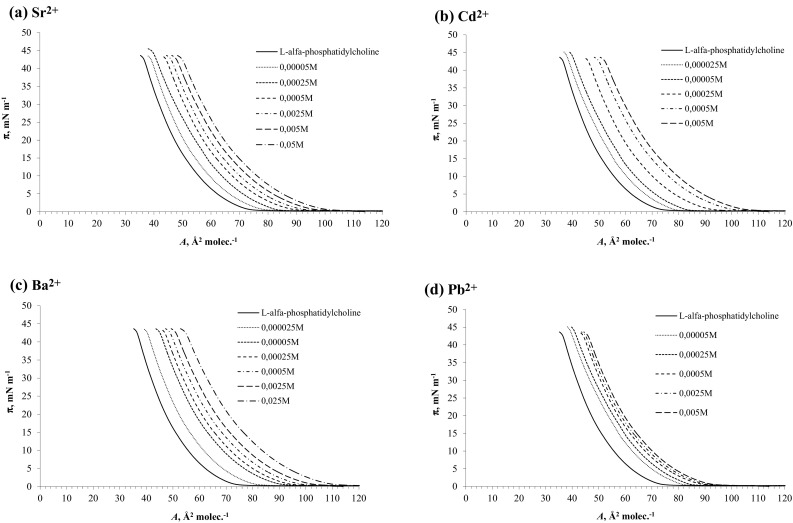
*π*–*A* isotherms of L-α-phosphatidylcholine from egg yolk monolayers in the absence of Me^2+^ ions (marked as a continuous line) and in the presence of ions in different concentrations: Sr^2+^ (**a**), Cd^2+^ (**b**), Ba^2+^ (**c**), and Pb^2+^ (**d**)

Figure 2a–d presents *π*–*A* isotherms of L-α-phosphatidylcholine from egg yolk monolayers in the absence of Me^2+^ ions (marked as a continuous line) and in the presence of Sr^2+^ (a), Cd^2+^ (b), Ba^2+^ (c), and Pb^2+^ (d). These isotherms are in satisfactory agreement with previously reported results [17, 37, 38]. The L-α-phosphatidylcholine from egg yolk monolayer is an example of a liquid-expanded membrane, with hydrophilic head groups located in the aqueous subphase and hydrophobic fatty acid tails oriented toward the air. The surface area per lipid molecule assumed various values depending on the length, conformation, and degree of unsaturation of the hydrocarbon chains. The surface area of the L-α-phosphatidylcholine from egg yolk molecule in pure water (56 Å^2^) was consistent with literature values [17, 37, 38]. Surface areas of L-α-phosphatidylcholine from egg yolk in the presence of Me^2+^ ions, for example at 0,0005 M a were as follows: 66 Å^2^ with Sr^2+^, 72 Å^2^ with Cd^2+^, 72.5 Å^2^ with Ba^2+^, and 65 Å^2^ with Pb^2+^. The limiting area of the isotherms depends on the presence of subphase ions. The addition of divalent ions to the subphase stabilized the monolayers and increased the limiting areas of the monolayer. The activity of the Pb^2+^ ion for causing changes in the surface properties of the films was much less than that observed for Ba^2+^, Cd^2+^ and Sr^2+^.

**Fig. 3:**
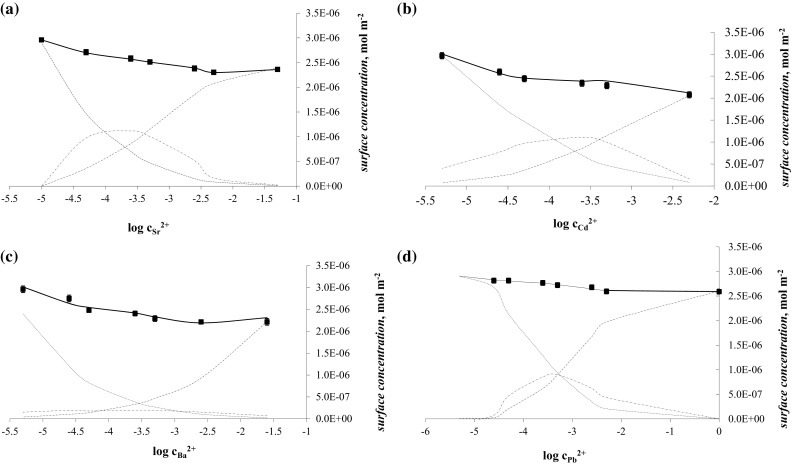
The dependence of total surface concentration of L-α-phosphatidylcholine from egg yolk, versus the logarithm of Me^2+^ ions concentration: Sr^2+^ (**a**), Cd^2+^ (**b**), Ba^2+^ (**c**), Pb^2+^ (**d**). (symbols for figures: filled square, the experimental values; continuous line, the theoretical curves; dashed line, $c_{\text{L}}$ form; dashed single dotted line, $c_{{{\text{LMe}}^{2 + } }}$ form and dashed double dotted line, $c_{{{\text{L}}_{2} {\text{Me}}^{2 + } }}$ form) at surface pressure ~40 mN m^−1^

Figure 3a–d presents the total surface concentrations of L-α-phosphatidylcholine from egg yolk versus the logarithm of the Me^2+^ concentration. Results obtained using Eq. (6) are presented by continuous lines, and surface concentrations of L-α-phosphatidylcholine from egg yolk–divalent ion membrane components are marked with broken lines. This figure represents the situation of a uniform distribution of monolayer components on the air/water interface of the lipid layer. From Eq. (6), the total surface concentration of the L-α-phosphatidylcholine from egg yolk membrane is the sum of the surface concentrations of its components (i.e., L, LMe^2+^, and L_2_Me^2+^).

Table 1 summarizes physicochemical parameters for monolayers of L-α-phosphatidylcholine from egg yolk and divalent ions (Mg^2+^ [20], Ca^2+^ [17], Sr^2+^, Cd^2+^, Ba^2+^, and Pb^2+^). Surface concentrations of individual components of the monolayer membrane were determined from Eqs. (14) to (16), by performing linear regression in Excel 2010. The $c{}_{{{\text{LMe}}^{2 + } }},c_{{{\text{L}}_{2} {\text{Me}}^{2 + } }}$ values were determined in this way for all Me^2+^ ions. Areas occupied by one L-α-phosphatidylcholine from egg yolk molecule, one LMe^2+^ complex, and one L_2_Me^2+^ complex (Me^2+^ = Sr, Cd, Ba, or Pb) were determined from Eqs. (9) to (10). Stability constants $K_{1}$ and $K_{2}$ were calculated by inserting experimental data into Eqs. (11) and (12), respectively.

**Table 1:** Physicochemical parameters for 1:1 and 1:2 L-α-phosphatidylcholine from egg yolk–divalent ion (Mg^2+^ [20], Ca^2+^ [17], Sr^2+^, Cd^2+^, Ba^2+^, and Pb^2+^) complexes

Examined ion (Me^2+^)	Mg^2+^ [20]	Ca^2+^ [17]	Sr^2+^	Cd^2+^	Ba^2+^	Pb^2+^
Calculated parameters						
$A_{{{\text{LMe}}^{2 + } }}$ (Ǻ^2^ molecule^−1^)	77 ± 0.77	65 ± 0.65	68 ± 0.68	69 ± 0.69	71 ± 0.71	73 ± 0.73
$A_{{{\text{L}}_{2} {\text{Me}}^{2 + } }}$ (Ǻ^2^ molecule^−1^)	109 ± 1.09	117 ± 1.17	120 ± 1.20	122 ± 1.22	122 ± 1.22	124 ± 1.24
$K_{1}$ (m^3^ mol^−1^)	9.95 × 10^2^	1.92 × 10^3^	2.88 × 10^3^	4.89 × 10^3^	3.46 × 10^3^	5.88 × 10^3^
$K_{{{\text{L}}_{2} {\text{Me}}^{2 + } }}$ (m^2^ mol^−1^)	3.87 × 10^4^	5.35 × 10^5^	6.42 × 10^4^	9.24 × 10^5^	7.70 × 10^5^	1.11 × 10^6^
Gibbs free energy of complexation (kJ mol^−1^)						
LMe^2+^	− 16.91 ± 0.51	− 18.52 ± 0.56	− 19.51 ± 0.58	− 20.81 ± 0.62	− 19.96 ± 0.60	− 21.26 ± 0.64
L_2_Me^2+^	− 25.88 ± 0.76	− 32.31 ± 0.97	− 27.12 ± 0.81	− 33.65 ± 1.01	− 33.21 ± 1.00	− 34.10 ± 1.02

Stability constants for the L-α-phosphatidylcholine from egg yolk–divalent ions (1:1) complexes ranged from 9.95 × 10^2^ for LMg^2+^ to 5.88 × 10^3^ for LPb^2+^. For the 2:1 complexes, these values ranged from 3.87 × 10^4^ for L_2_Mg^2+^ to 1.11 × 10^6^ for L_2_Pb^2+^. Surface areas occupied by the L-α-phosphatidylcholine from egg yolk–divalent ions (1:1) complexes ranged from 65 ± 0.65 Å^2^ molecule^−1^ for LCa^2+^ [17] to 77 ± 0.77 Å^2^ molecule^−1^ for LMg^2+^ [20]. For L_2_Me^2+^ (2:1) complexes, the range was 109–124 ± 0.12 Å^2^ molecule^−1^.

Complexes containing Pb^2+^ ions had the largest stability constant and surface area values among the L-α-phosphatidylcholine from egg yolk–divalent ion complexes. The excellent agreement between the experimental and theoretical points validated the assumption that LMe^2+^ and L_2_Me^2+^ complexes formed between the L-α-phosphatidylcholine from egg yolk monolayer and divalent ions. The calculated area of one L-α-phosphatidylcholine from egg yolk molecule from Eq. (8) was the same as the experimental value (56 Å^2^ molecule^−1^) obtained from the Langmuir method (Fig. 1). Areas occupied by one L_2_Me^2+^ complex were smaller than the sums of the surface areas of the complex components. This result is probably related to the arrangement of L-α-phosphatidylcholine from egg yolk molecules in the complexes and the structural construction of the complexes. The relatively high stability of LMe^2+^ and L_2_Me^2+^ complexes provided evidence supporting the prevalence of 1:1 and 2:1 complexes in L-α-phosphatidylcholine from egg yolk monolayers in the presence of Me^2+^. In our opinion, the Me^2+^ ions interact with a head-group moiety, most likely the phosphate group.

Gibbs free energy values presented in Table 1 (calculated from Eq. 17) for all complexes ranged from 17 to 21 kJ mol^−1^ for the 1:1 complexes and from 26 to 34 kJ mol^−1^ for the 2:1 complexes.

**Fig. 4:**
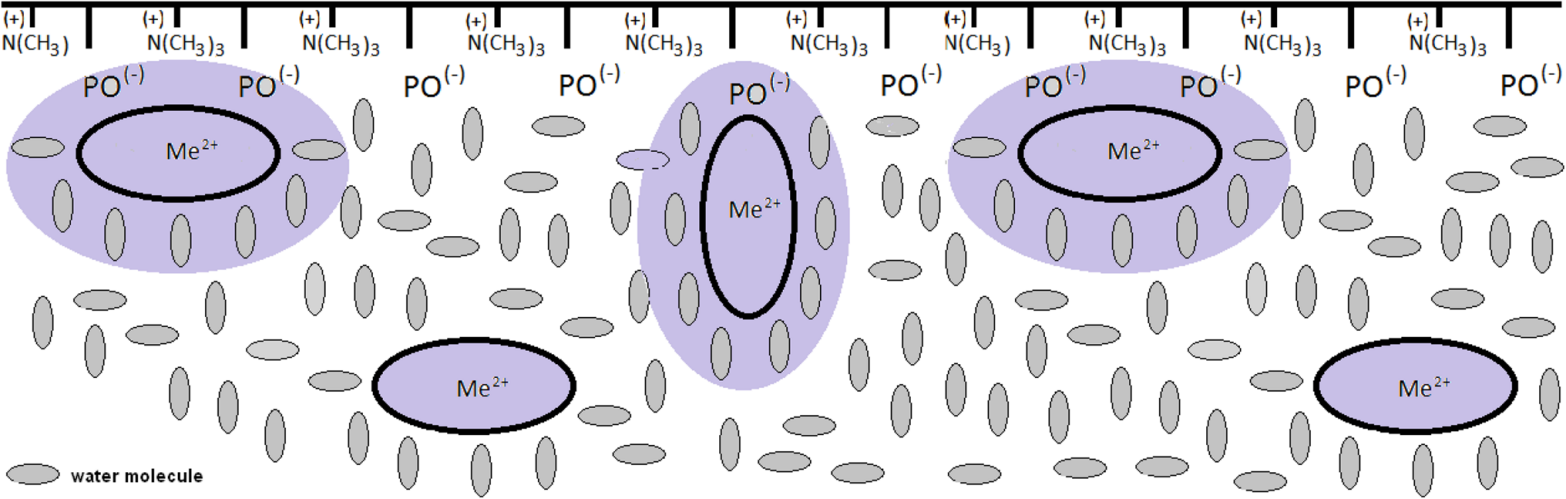
Schematic representation of L-α-phosphatidylcholine from egg yolk monolayer with divalent ions (LMg^2+^ and L_2_Mg^2+^ complexes formation)

Figure 4 presents the schematic diagram of LMe^2+^ and L_2_Mg^2+^ complex formation. Positively charged divalent ion is able to bind electrostatically to the negatively charged groups in monolayer membranes. The binding to membrane phospholipid head-groups may change the local conformation and have a general electrical screening effect [39]. In our opinion the Me^2+^ ion interacts with the head-group moiety, most likely the phosphate group. Divalent ion and phosphate are known to form a strong ion pair in water and the strength of this interaction is likely to be increased in the lipid head-group region where the dielectric permittivity of the surrounding (and thus the electrostatic screening of charges) is reduced.

Using the areas occupied by the L, LMe^2+^, and L_2_Me^2+^ monolayer components and the stability constants of the complexes, we calculated theoretical values of the surface concentrations of the L-α-phosphatidylcholine from egg yolk monolayers in the presence of Me^2+^ ions from Eq. (6). We obtained very good agreement between the theoretical and experimental values (lines and points, respectively, in Fig. 3a–d). This finding further verified the assumption that LMe^2+^ and L_2_Me^2+^ complexes formed in the lipid monolayer.

## Conclusions

The interactions of both components on a monolayer are modulated extrinsically by mobile ions in the surrounding medium. As a continuation of our study [17, 20], this article examines the effect of divalent cations (Me^2+^) on L-α-phosphatidylcholine from egg yolk monolayers. We studied lipid–ion interactions as a function of divalent ion concentrations. Here, we present evidence for the formation of LMg^2+^ and L_2_Mg^2+^ complexes at the air/water interface and calculate their stability constants, surface areas and Gibbs free energy. The knowledge of stability constants of L-α-phosphatidylcholine from egg yolk–Me^2+^ system let us understand the processes that take place both in the monolayer itself and also on its surface.

The data presented in this work are of great importance for the interpretation of phenomena occurring in lipid monolayers and bilayers, especially the effects of divalent ions. The simple and very interesting methods proposed in this paper and in earlier studies may be used with success to determine the lipid–divalent ion equilibria in the lipid monolayer.

In conclusion, we would like to emphasize that the stability constants for L-α-phosphatidylcholine from egg yolk and divalent ions complexes in monolayers have been reported here for the first time.
